# Multi-level factors influence the implementation and use of complex innovations in cancer care: a multiple case study of synoptic reporting

**DOI:** 10.1186/s13012-014-0121-0

**Published:** 2014-09-16

**Authors:** Robin Urquhart, Geoffrey A Porter, Joan Sargeant, Lois Jackson, Eva Grunfeld

**Affiliations:** Department of Surgery, Dalhousie University, Halifax, Nova Scotia Canada; Cancer Outcomes Research Program, Dalhousie University/Capital Health, Halifax, Nova Scotia Canada; Department of Community Health and Epidemiology, Dalhousie University, Halifax, Nova Scotia Canada; Division of Medical Education, Dalhousie University, Halifax, Nova Scotia Canada; Continuing Professional Development, Dalhousie University, Halifax, Nova Scotia Canada; School of Health and Human Performance, Dalhousie University, Halifax, Nova Scotia Canada; Atlantic Health Promotion Research Centre, Dalhousie University, Halifax, Nova Scotia Canada; Ontario Institute for Cancer Research, Toronto, Ontario Canada; Department of Family and Community Medicine, University of Toronto, Toronto, Ontario Canada

**Keywords:** Synoptic reporting, Knowledge translation, Implementation, Cancer, Innovation

## Abstract

**Background:**

The implementation of innovations (*i.e.*, new tools and practices) in healthcare organizations remains a significant challenge. The objective of this study was to examine the key interpersonal, organizational, and system level factors that influenced the implementation and use of synoptic reporting tools in three specific areas of cancer care.

**Methods:**

Using case study methodology, we studied three cases in Nova Scotia, Canada, wherein synoptic reporting tools were implemented within clinical departments/programs. Synoptic reporting tools capture and present information about a medical or surgical procedure in a structured, checklist-like format and typically report only items critical for understanding the disease and subsequent impacts on patient care. Data were collected through semi-structured interviews with key informants, document analysis, nonparticipant observation, and tool use/examination. Analysis involved production of case histories, in-depth analysis of each case, and a cross-case analysis. Numerous techniques were used during the research design, data collection, and data analysis stages to increase the rigour of this study.

**Results:**

The analysis revealed five common factors that were particularly influential to implementation and use of synoptic reporting tools across the three cases: stakeholder involvement, managing the change process (*e.g.*, building demand, communication, training and support), champions and respected colleagues, administrative and managerial support, and innovation attributes (*e.g.*, complexity, compatibility with interests and values). The direction of influence (facilitating or impeding) of each of these factors differed across and within cases.

**Conclusions:**

The findings demonstrate the importance of a multi-level contextual analysis to gaining both breadth and depth to our understanding of innovation implementation and use in health care. They also provide new insights into several important issues under-reported in the literature on moving innovations into healthcare practice, including the role of middle managers in implementation efforts and the importance of attending to the interpersonal aspects of implementation.

**Electronic supplementary material:**

The online version of this article (doi:10.1186/s13012-014-0121-0) contains supplementary material, which is available to authorized users.

## Background

In cancer care, therapeutic decisions are often based on input from a multidisciplinary team of specialist physicians [1]. For patients with suspected or confirmed cancer, clear recordings of diagnostic and surgical procedures and findings support accurate diagnosis and staging, and facilitate treatment planning. The dominant method of reporting such findings is the narrative report, which is a free text, descriptive account of the procedure, findings, and proposed treatment. Research has demonstrated, across settings and diseases, these types of reports inconsistently and incompletely provide the information required to understand the disease and make informed care decisions [2-7].

Another method of reporting, the synoptic report, captures and presents information in a structured, checklist-like manner and typically reports only items critical for understanding the disease and subsequent impacts on patient care. Research has consistently demonstrated that synoptic reports greatly improve the quality of pathology [1,2,8-19] and surgical [5,20-24] reporting. When electronic, synoptic reporting tools (SRTs) result in health system efficiencies [24-26] and provide an effective mechanism to generate real-time data [20,25,27,28]. They have been widely endorsed as a means of standardizing cancer reporting, and improving the availability and quality of clinical information for persons diagnosed with cancer [29-33]. However, SRTs represent complex innovations (*i.e.*, new knowledge, tools, or practices), with their implementation and use requiring changes in clinical practice [34] and support from the organization and larger healthcare system.

Much of healthcare delivery occurs as part of a team within a complex organizational structure that is situated in a historical, cultural, economic, and political context. Thus, the setting in which an innovation is implemented, the timing of implementation and the cultural, economic, and socio-political climate during that particular period of time, and the individuals involved may all affect whether, and the extent to which, new ideas and tools are implemented in clinical practice. Researchers increasingly acknowledge the important role these contextual factors play in innovation implementation and use in health care [35-39]. The objective of this study was to examine the key interpersonal, organizational, and system level factors (hereafter referred to as ‘multi-level’ factors) that influenced the implementation and use of SRTs in three cases of cancer care. Each case represented a SRT initiative in a particular clinical area.

## Methods

The methods are described in detail elsewhere [40] and presented briefly here. Case study methodology [41,42], employing an explanatory multiple-case design, was used to explore which factors were important to SRT implementation and use, examine relationships amongst factors, and uncover which factors appear to be similar (and distinct) across cases. Four units of analysis were attended to within each case: the implementation team, the clinician user(s), the organization (hospital), and the broader health system (sociopolitical, historical, and regulatory context). The study was approved by the Research Ethics Boards at all applicable institutions.

### Theoretical perspectives

Three theoretical perspectives largely informed the design of this study:

1. Promoting Action on Research Implementation in Health Services (PARiHS) framework [43,44]
2. Organizational framework of innovation implementation [45]
3. ‘Systems’ thinking / change [46]

Table 1 provides a brief description of each theoretical perspective. When taken together, these perspectives presented a range of interpersonal, organizational, and system influences on practice change and therefore identified potentially important factors to study. During study design, they informed case selection by helping identify cases that appeared to vary in terms of potentially important factors. During data collection, they informed which potential key informants were approached, which documents were sampled, and the questions posed to key informants during the in-depth interviews.

**Table 1:** **Brief descriptions of the theoretical perspectives used in this study**

**Theoretical perspective**	**Description**
Promoting action on Research Implementation in Health Services (PARiHS) framework	The PARiHS framework, which has undergone continual refinement since its initial publication in 1998 [43], proposes the implementation of evidence into practice is a function of the interaction between three core elements: 1) the level and nature of evidence; 2) the context or setting into which evidence is implemented (with context consisting of the sub-elements of culture, leadership, and evaluation); and 3) the method by which the process is facilitated. These elements are conceptualized as existing on a continuum, with high evidence, context, and facilitation driving successful implementation.
Organizational framework of innovation implementation	Helfrich and colleagues [45] adapted an organizational model on the implementation of complex innovations from the manufacturing sector [47,48] for healthcare settings. This adapted framework comprises the following six elements and highlights relationships amongst these elements: management support, financial resource availability, implementation policies and practices, implementation climate, innovation champions, and innovation-values fit. The authors posit that these elements play important roles in achieving implementation effectiveness (*i.e.*, consistent, committed, and skilled innovation use [47]).
‘Systems’ thinking/change	Kitson [46] reviewed the critical social science, action science, diffusion and management of innovations, practice development, and learning organizations and systems theories literature to explore the underlying assumptions and theories used to describe how knowledge is translated into practice. The resulting critique posits that the successful translation of knowledge into practice is a function of: 1) how the individuals involved understand the nature and characteristics of the new knowledge; 2) their level of autonomy in making decisions about using the new knowledge; 3) how they negotiate and renegotiate relationships with others in the system; and 4) how they attract the resources needed to sustain changes in practice (whereby the involvement of key stakeholders is deemed critical to controlling and attracting resources).

### Sampling

Using the sampling guidance of Yin [42] and Stake [41], three cases in Nova Scotia, Canada, were selected for study. Nova Scotia is a small Canadian province, with a population of approximately 940,000. Health care is delivered through nine health regions and the province’s consolidated women’s and children’s hospital. Information with respect to potential cases was obtained prior to case selection, via publicly available documents and discussions with implementation leaders, to guide sampling decisions. The three cases selected were:

1. Synoptic reporting in the Nova Scotia Breast Screening Program (hereafter referred to as the ‘mammography case’);
2. Synoptic reporting in the Colon Cancer Prevention Program (hereafter referred to as the ‘endoscopy case’); and
3. Synoptic reporting in the Surgical Synoptic Reporting Tools Project (hereafter referred to as the ‘cancer surgery case’).

Though differences existed, two cases overlapped with respect to settings, timing, and individuals involved (endoscopy case, cancer surgery case), while one case differentiated considerably in terms of these contextual conditions (mammography case). Based on existing, pre-study knowledge, these cases were also perceived to converge and diverge with respect to many factors that, based on the theoretical perspectives and broader literature, were likely to influence the implementation and use of an innovation in clinical practice.

### Data collection procedures

Multiple data collection procedures were used to gain rich, detailed information about each case and to increase the likelihood of achieving data triangulation across informants, units of analysis, and data collection methods [49,50]:

1. One-on-one semi-structured interviews [51] were conducted with key informants at the different units of analysis. Key informants were identified through purposive and snowball sampling. Interview questions were adapted based on each case’s unique context as well as the person being interviewed and his/her role in the implementation. One researcher (RU) conducted all interviews. Each interview was audio-recorded, transcribed verbatim, and checked for accuracy. Following each interview, the questions and responses were reviewed to determine whether the issues were answered in sufficient depth and whether the questions or probes needed revisions [52]. Any revisions to the script were completed prior to the next interview.
2. Documents were analyzed for each case. They were acquired from the implementation teams, other key informants, and Internet searches. Documents included project plans/charters, team/organizational reports, formal/informal evaluations, and communications materials, and records related to the structure, infrastructure, and governance of Nova Scotia’s health system. All documents were reviewed to gain an historical and contextual perspective on the initiative and to corroborate and augment evidence from other sources [42].
3. Non-participant observation [51] was conducted to observe training sessions related to use of the surgical tool and initial surgeon reactions to viewing/using the SRT. These sessions were conducted for one case only (cancer surgery) since the implementation of this SRT was occurring during data collection.
4. Each SRT was examined to gain insight into the technical operations related to using the system. Final synoptic reports, with patient identifying information concealed, were reviewed to examine content and format.

Field notes were also taken during and following interviews, observation sessions, and examination of the SRTs. One researcher (RU) attempted to resolve any inconsistencies and contradictions across sources and methods through re-reviewing transcripts/documents and further inquiry (*e.g.*, follow-up with informants).

### Analysis

Analysis commenced with the first data collected. One researcher (RU) constructed detailed case descriptions for each case to describe the history, context, and organization of the initiative, and the SRT that was implemented.

The three cases were treated as separate studies and analysed independently. A thematic analysis was conducted for each case [53], involving coding, collating codes, and generating, reviewing, and refining themes. A coding framework was developed during a pilot study [54], with subsequent minor refinements until no new concepts emerged. All interview transcripts and field notes were coded line-by-line in their entirety. Coding was performed manually by labeling the code in the margin of a hard copy of the transcript. Documents were read (and re-read) to identify contextual/historical data, record concepts/codes and link them to specific document excerpts, and triangulate findings from other data sources.

The collapsing of codes into categories and the identification and refinement of themes involved iterative processes between the case-specific data and the theoretical perspectives as well as other literature sources [55-58]. These processes included constructing tables that identified emerging categories and themes in relation to the three theoretical perspectives in order to understand how, and the extent to which, the case-specific data aligned (or not) with the constructs of the theoretical perspectives. While the theoretical perspectives helped guide the study, the researcher made concerted efforts to seek out conflicting evidence and to examine whether other factors were key facilitators or enablers of SRT implementation and use, such as those described in psychological and behavioral theories [59-61]. Emergent findings were discussed on multiple occasions with the research team to assist the analytic process and questioning of the data.

The final stage of analysis was a cross-case analysis to compare and contrast themes across cases. Each similar theme was examined in regards to the ‘direction of influence’ the theme took in the context of each case. For instance, one overarching theme—or key factor—may have been a facilitator in one case due to its presence, but a barrier in another case due to its absence. Divergent themes were examined in regards to their specific importance to the particular case/context.

Numerous techniques were used during research design, data collection, and analysis to increase the rigour of this study. These are presented in Table 2.

**Table 2:** **Techniques employed to increase rigour**

**Phase**	**Method**
Research design	Use of multiple theoretical perspectives to guide research design, analyses, and interpretation, helping to build a wider explanation of the phenomenon and a means of exploring a range of plausible theoretical interpretations [49,50,62].
	Strategic selection of three cases to support greater confidence in findings. This strategy included selecting cases based on replication logic [42] and to provide good learning opportunities [41].
	Pilot work [54] to refine data collection and analyses processes, including the development of a coding framework and refinement of interview guides, and inform the final study design.
Data collection	Use of key informants across four units of analysis (individual user, implementation team, organization, and larger system) and multiple data collection methods. This allowed researchers to uncover converging findings across informants, units of analysis, and data collection methods (*i.e.*, triangulation) [49,50].
Analysis	Maintaining a case study database [42] consisting of a complete set of all the data collected for each case and all records related to the treatment of the data during the analytic process.
	Considering other plausible explanations for the findings and seeking out additional evidence where inconsistencies or contradictions existed. Both helped minimize the confirmation of preconceived ideas [42,63] and the possibility that the researcher selectively described and explained the events to support a favoured theory or perspective.
	Maintaining a chain of evidence [42] throughout data analysis (often referred to as an audit trail). This involved documenting an explicit trail that identified the links between the data collected and the interpretations/conclusions.
	Member checking to verify specific factual data and to ask participants for their responses/reactions to findings.
	Multiple meetings/discussions of the research team to review the analytic procedures and discuss and question the findings.

## Results

Table 3 presents key informant participation for this study by case and unit of analysis. Six individuals invited to partake in the mammography case did not respond; one individual invited in the endoscopy case did not respond; and no individuals invited in the cancer surgery case did not respond. However, one surgeon in the cancer surgery case refused to be observed during training. All individuals who did not respond were clinician users. Two informants participated in two interviews (*e.g.*, initial and follow-up interviews). Table 4 presents the documents reviewed for each case.

**Table 3:** **Key informant role and setting (if applicable), by unit of analysis**

	**Case A: Mammography case**	**Case B: Endoscopy case**	**Case C: Cancer surgery case**
**Implementation team** ^**a**^	Team member #1	Team member #1	Team member #1
	Team member #2	Team member #2	Team member #2
	Team member #3	Team member #3	Team member #3
		Team member #4	
	*3*	*4*	*3*
**Clinician users** ^**b**^	Physician, tertiary^c,d^	Physician, tertiary^c,d^	Physician, tertiary
	Physician, community	Physician, tertiary	Physician, tertiary
	Physician, community	Physician, tertiary	Physician, tertiary
	Physician, community	Physician, community^d^	Physician, tertiary
		Physician, community	Physician, community^d^
			Physician, community
	*4*	*5*	*6*
**Organization**	Department head, tertiary	Department head, tertiary	Manager, tertiary
	Department head, community	Manager, tertiary	Manager, tertiary
	Manager, community	Manager, community	Manager, tertiary
	Manager, community	Manager, community	Manager, community
	Report end user, tertiary	Report end user, tertiary	Report end user, tertiary
			Report end user, tertiary
			Report end user, tertiary
	*5*	*5*	*7*
**System**	Health district CEO	Health district CEO	Health district CEO
	Executive, Department of Health	Executive, Department of Health	Executive, Department of Health
	Manager, provincial service organization	Executive, provincial program	Executive, provincial program
		Executive, provincial program	Executive, provincial program
		Manager, provincial service organization	Manager, provincial service organization
	*3*	*5*	*5*

^a^All implementation team members in all cases were located in tertiary care settings.

^b^Clinician users in the mammography case were radiologists; clinician users in the endoscopy case were endoscopists (gastroenterologists and surgeons); clinician users in the cancer surgery case were surgeons.

^c^Heavily involved in initial tool design and ongoing refinement.

^d^Identified by other key informants as a local physician champion.

**Table 4:** **Documents collected and reviewed**

	**Source**	**Type**
Case A: Mammography case	Web search	Annual reports, from 2005-2011
		Research/conference presentations (2 PowerPoint [PPT] documents)
		Communications materials (press release, newsletter) (2 documents)
		Media article (1 document)
	Implementation team	Sample synoptic reports
		History/timeline (PPT slides)
		Schematic of program and its processes/procedures (PPT slides)
		Article: professional journal (1 document)
Case B: Endoscopy case	Web search	Communications materials (*e.g.*, press releases, newsletters, communications briefs) (6 documents)
		Report on population-based colorectal cancer screening in Nova Scotia (1 document)
		Provincial practice recommendations (1 document)
		National position statements (2 documents)
		Report on colorectal cancer screening in Canada (1 document)
		Program/strategy elements of Canadian colorectal cancer screening programs (1 PPT file)
		Quality determinants of Canadian colorectal cancer screening programs (1 PPT file)
		Requirements/gap analysis of software applications (1 document)
	Implementation team	Sample synoptic reports
		Implementation strategy (1 document)
		Provincial evaluation (1 PPT file)
		Public presentation (1 PPT file)
	Other key informants	Professional association published consensus guidelines (1 document)
		Media article (1 document)
Case C: Cancer surgery case	Web search	Communications materials (press release, 2 newsletters) (3 documents)
		Conference presentation (1 PPT file)
	Implementation team	Sample synoptic reports
		Project charter (1 document)
		Lessons learned (1 document)
		Presentation from national conference (1 PPT file)
		Presentation to local stakeholders (1 PPT file)
	Other key informants	Funder implementation strategy/directions (4 PPT presentations)
		Funder evaluation (1 document, 1 PPT file)
		(Inter)national List Serve discussion on synoptic reporting (all emails over 1 month period)
System context	Web search	Reports/discussion papers on privacy and personal health information legislation (3)
		Acts on privacy/personal health information, Nova Scotia (4)
		Act on privacy/personal health information, Federal (1)
		Pan-Canadian framework on privacy/personal health information (1)
		Hospital Business Plans (2)
		Consultant’s report on Nova Scotia’s healthcare system (1)
		Report/review on Nova Scotia’s E-health system (1)
		Journal article on Nova Scotia’s E-health system (1)
		Cancer Management Strategy for Nova Scotia (1)
		Evaluation of Cancer Care Nova Scotia (1)

### Case descriptions

Table 5 provides brief descriptions of each of the cases. A detailed description of the broader healthcare system is presented in an online appendix.

**Table 5:** **Synoptic reporting tool (SRT) implementation in each of the cases**

**Case**	**Description**
Nova Scotia Breast Screening Program	Synoptic mammography reporting began in the mid-1980s at one academic hospital. The impetus was to develop a database that facilitated radiologists’ abilities to track patients subsequent to suspicious imaging to ensure they received appropriate and timely follow-up care. The initiative was started as a research project, with funds from a local research foundation to purchase computing software and hardware. One individual developed a diagnostic SRT with self-taught computing skills. Within a few years, it was implemented at a nearby community hospital. At the time, the concept of synoptic reporting was unprecedented, with the developer having no knowledge of a similar system nationally or internationally. After establishment of the Nova Scotia Breast Screening Program as a provincial program in 1991, the program developed and implemented a similar SRT to report and capture data on all screening mammography in the province. The Nova Scotia Breast Screening Program also became the host of the diagnostic SRT, essentially creating one system to capture all mammography (screening and diagnostic) in Nova Scotia. The capabilities and functions of these SRTs position them somewhere in the middle of the evolution of synoptic reporting technology [1]. The end report that is generated is not synoptic in nature, but rather consists of a series of standardized paragraphs, separated by structured headings, which reads similar to a traditional narrative report.
	Though the Nova Scotia Breast Screening Program hosted and operated these SRTs, it could not mandate their implementation and use in individual hospitals across the province. As a consequent, their expansion across the province occurred in a gradual, largely unplanned, manner. By October 2008, all hospitals in the province had implemented the screening SRT. This was in response to a governmental policy established several years earlier related to screening mammography standards. By 2010, the diagnostic SRT had been implemented at all diagnostic imaging departments in the province that perform mammography, yet, at the time of this study, radiologists in three health districts continued to refuse to use this SRT to report diagnostic mammography.
Colon Cancer Prevention Program	Synoptic colonoscopy reporting was implemented with the rollout of the Colon Cancer Prevention Program,^a^ beginning in Spring 2009. The impetus for including synoptic reporting in the program was quality improvement, with leaders believing that measurement was critical to improving colonoscopy performance and to following up participants in the screening pathway. The endoscopy reporting software and database from the Clinical Outcomes Research Initiative (CORI), developed at Oregon Health and Science University, was selected as the SRT. The application was modified as little as possible, though some customization was necessary. The software’s capabilities positioned CORI at the advanced end of synoptic reporting technology [1]. The final report is in narrative form: although the data are entered synoptically, CORI takes the responses and creates them into standard sentences and paragraphs.
	SRT implementation was phased in over a two-year period across the entire province (nine health districts) and funded by the provincial Department of Health. To participate in the Colon Cancer Prevention Program and perform screening colonoscopy (the recommended investigation following a positive fecal immunochemical test), endoscopists were required to sign an agreement stating they would use the SRT for all colonoscopies, screening and diagnostic, with the goal of having a single database capturing all colonoscopy in the province. Funding arrangements ensured that endoscopists used the SRT for screening colonoscopy—they would not get paid for these procedures otherwise. However, by the end of data collection, endoscopists in most districts were not using the SRT for diagnostic colonoscopy. The reason provided by most endoscopists was the lack of integration with existing hospital information technology systems,^b^ leading to additional work for endoscopists and endoscopy unit staff.
Surgical Synoptic Reporting Tools Project	Surgical synoptic reporting was implemented in Nova Scotia between 2010–2011 at three hospitals (two academic, one community). The Surgical Synoptic Reporting Tools Project began as a pilot project for breast and colorectal cancer surgery, funded and led by the Canadian Partnership Against Cancer, a national organization leading the implementation of Canada’s cancer control strategy. The project was based on the successful development and implementation of synoptic reporting for cancer surgery in one Canadian province, which led to a national collaboration to expand surgical synoptic reporting to other Canadian jurisdictions. The SRT was the Web-based Surgical Medical Record (WebSMR), originally developed in Alberta [25]. The WebSMR was adapted to meet provincial and local hospital contexts. Its features and capabilities placed WebSMR at the cutting edge of synoptic reporting technology [1]. The final operative report is synoptic in nature, presented in a checklist-like format. The tool was fully integrated with each hospital’s existing information technology systems, allowing seamless transfer of information across systems, including transfer of the final operative report into the patient’s electronic medical record immediately on completion.
	As a pilot project, a small number of surgeons (nine) were selected to participate across disease sites and hospitals. Planning and implementation occurred over a 3.5-year time period. The team had neither the authority to mandate SRT use nor the capacity to influence use through organizational or provincial policies.

^a^The Colon Cancer Prevention Program is the provincial population-based colorectal cancer screening program.

^b^By the end of data collection (Winter 2012), the SRT was interfaced with hospital information technology (IT) systems in one health district, allowing seamless transfer of information (*e.g.*, patient demographics, colonoscopy report) across systems (*e.g.*, patient registration systems, electronic medical records). For the remaining eight districts, the SRT was not interfaced with existing hospital IT systems (the work to complete this goal was ongoing) and a variety of interim processes were used to transfer the colonoscopy report to the patient’s medical record.

Additional file 1.

### Factors influencing implementation and use

Table 6 presents both the common and distinct factors influencing the implementation and use of each SRT and specifies the direction of influence (facilitating or impeding) for each case. One factor related to characteristics of the SRT itself and was conceptualized as existing at the level of the innovation. Five common factors were particularly influential to SRT implementation and use across the three cases: stakeholder involvement, managing the change process, administrative and managerial support, champions and respected colleagues, and innovation attributes.

**Table 6:** **Common and distinct factors influencing synoptic reporting tool (SRT) implementation and use across cases**

**Factor**	**Case A: Mammography case**	**Case B: Endoscopy case**	**Case C: Cancer surgery case**
*Common factors*			
Stakeholder involvement	+/− Initial implementation and use were facilitated by stakeholder involvement; subsequent expansion was impeded by low stakeholder (*i.e.*, radiologist) involvement	- Implementation was impeded by limited stakeholder involvement	+ Implementation was facilitated by early, ongoing, and collaborative stakeholder involvement
Managing the change process	- Implementation and use were impeded by sub-optimal change management practices	- Implementation and use were impeded by sub-optimal change management practices, though user training was well conducted	+ Implementation and use were facilitated by high-quality change management practices
Administrative and managerial support^a^	+/− Implementation was facilitated by high administrative support and high managerial support in some hospitals; implementation was impeded by low managerial support in other hospitals	+/− Implementation was facilitated by high administrative support; implementation was impeded by low managerial support in many hospitals	+ Implementation was facilitated by high administrative and managerial support
Champions and respected colleagues	+/− Implementation and use were facilitated by clinical and administrative champions; lack of clinical champions in some districts impeded use	+ Implementation and use were facilitated by clinical champions and respected clinical colleagues	+ Implementation and use were facilitated by clinical champions and respected clinical colleagues
Innovation attributes	+/− Implementation and use were facilitated by alignment with individuals’ and organizations’ values, interests, and needs; use was impeded by perceived tool (and final report) deficiencies and its relative (dis)advantage in practice	+/− Use was facilitated by the tool’s perceived ease of use, but impeded by IT and other technical issues; implementation and use were facilitated by alignment with individuals’ and organizations’ values, priorities, and interests	+/− Use was facilitated by the tool’s perceived ease of use, but impeded by accessibility and IT issues; implementation and use were facilitated by alignment with individuals’ and organizations’ values, priorities, and interests
*Distinct factors*			
Implementation approach	NA	+ Implementation and use were facilitated by the tool’s positioning in the provincial screening program (however, the top-down, policy driven approach was met with much resistance)	+/− Implementation was facilitated by the tool’s positioning as a pilot project; use was impeded by its positioning since the team had no authority to influence use (*e.g.*, through policy)
Project management	NA	- Implementation was impeded by suboptimal project management, specifically related to the tool’s implementation	NA
Resources	- Implementation and use were impeded by insufficient resources for SRT development/updates, implementation, and expansion	NA	- Implementation was impeded early in the project by insufficient IT resources
Culture	+ Implementation and use were facilitated by the program’s strong quality improvement culture; however, this strong culture was viewed negatively by some users, possibly influencing expansion	NA	NA
Leadership	+ Implementation and use were facilitated by consistent, effective leadership	NA	NA
Monitoring and feedback mechanisms	+ Implementation and use were facilitated by ongoing monitoring and feedback mechanisms	NA	NA
Components of the healthcare system	NA	- Implementation was impeded by structural, infrastructural, and socio-historical components of the healthcare system	- Implementation was impeded by relational and infrastructural components of the healthcare system

Depending on the context, the factor was a facilitator or barrier to implementation and use; + indicates a facilitating influence, − indicates an impeding influence.

NA = not applicable.

^a^Administrators = executive officers, directors, and senior management at the Department of Health, health district, and hospital levels; management = managers and heads of organizational departments and units.

### Stakeholder involvement

The breadth, depth, and timing of stakeholder involvement were critical to SRT implementation and use. This was found across cases (and across settings within cases) wherein the early, collaborative involvement of a broad range of stakeholders, including clinician users, managers and staff of relevant departments, and hospital/health region administrators, allowed implementation team members to develop and maintain relationships necessary to implement the SRTs within the various governance/regulatory structures and IT infrastructure, and to promote a sense of ownership amongst local stakeholders. Conversely, where stakeholder involvement was low, interview and documentary data revealed that this was a barrier to effective implementation and use.

In the endoscopy case, for instance, while the implementation team included key stakeholders as members of working groups and worked with local implementation teams, most key informants perceived a low level of involvement and many endoscopists stated their input was not requested at any point throughout SRT implementation. Limited stakeholder involvement and perceptions that their concerns were not always acknowledged led some key informants to describe unsatisfactory relationships with the implementation team, which some believed contributed considerably to some of the challenges the implementation team encountered during implementation, including the inability to integrate the SRT with existing IT systems in most hospitals: ‘[w]e probably would be all integrated right now had [the implementation team] actually heard the need’ (Organizational member #3). Data from the mammography case demonstrated high stakeholder involvement during SRT development and early implementation, but much less involvement during expansion, which has proven challenging for that case. As one clinician user stated, ‘[the implementation team] get[s] out there and realize[s] that no one else likes it because no one was involved in the development’ (Physician #2).

In contrast, key informant and document data from the cancer surgery case indicated that stakeholders viewed themselves as partners in the project, with their input sought and incorporated into the project planning as much as possible. Nearly all system and organizational members expressed high satisfaction with their depth of involvement and the implementation team’s responsiveness to their feedback and recommendations:

‘I thought that from a coding perspective, they were receptive to anything that we had to say and we certainly had lots of one-on-ones with [Dr. X] and said, ‘this is the challenge, this is what we think is missing, this is what we need to be clear on in terms of breast conservation versus mastectomy’ … [They were] more than receptive to take our concerns, our input, and then offer solutions or feedback’ (Organizational member #2).

Consequently, local implementations of this SRT were viewed as collaborative processes, the significance of which was emphasized by one implementation team member:

‘It was about … listening to [our partners] and respecting what they are saying. [Their] language and voice is reflected in the work. Without doubt, that is the number one thing that has made this successful …’ (Team member #2).

### Managing the change process

Across all cases, managing the people and processes involved in the change process was fundamental to SRT implementation and use. This involved building a case for SRTs, communicating about the change process (including articulating the value of the tools and how they fit into the ‘bigger picture’ of cancer care), equipping people to use the SRTs, and managing barriers and providing incentives to change.

In two of the cases (mammography case, endoscopy case), the data suggested that SRT implementation and use were negatively impacted by sub-optimal change management practices. Key informants perceived that communication about the SRTs and their implementation was inadequate. In the mammography case, several key informants stated they received no communication whatsoever about the tools prior to their implementation and many felt that implementation processes were hampered because users (and other organizational members) had a limited understanding of how the SRTs aligned with breast health/care in Nova Scotia:

‘I think that part of the problem was that people didn’t really understand how it fit into the grand scheme of things and, uhm, perhaps if [the implementation team] had been able to get that concept across better, [radiologists] would have been more accepting’ (Physician #1).

Similarly, in the endoscopy case, key informant interviews with organizational managers suggested that they did not perceive the value of the SRT. In the cancer surgery case, all key informants perceived value in the tool and understood the desired endpoint, suggesting that the implementation team was successful in communicating the reasons for SRT implementation: ‘My experience has been they know what they are doing, they know where they need to go and want to go, uhm, and continuing to make those strides with their colleagues’ (System member #2). Moreover, data from all cases suggest that much of the *effective* communication—*i.e.*, communicating the reasons for the change, how individuals’ work will be impacted, and what is expected of them during and after implementation—occurred through personal contact rather than formal communication channels.

Implementation teams from all cases provided initial, onsite training for clinician users and some level of ongoing technical support during and following implementation. The training environments and experiences differed across cases, with some users describing the training and support processes as challenging while others were pleased with the quality of training. Following initial training, the availability of ongoing support was critical to realizing committed, sustained SRT use. For instance, many endoscopists in the endoscopy case expressed frustration with ongoing support mechanisms and the timeliness of support processes whereas most surgeons in the cancer surgery case were particularly pleased with the high level of support provided early in implementation, specifically the 24/7 telephone access to a technical support person, as well as the ongoing support process.

The importance of an early positive implementation experience, especially with training and support, was highlighted across cases and sources of evidence. In the mammography case, when reflecting upon early training and support experiences, one informant stated that ‘the initial frustration was such that it was a deal breaker’ (Physician #4). In the cancer surgery case, interview, observation, and documentary evidence suggested that ‘… training should not be underestimated. In fact, the more training provided the better the implementation experience was*’* (Excerpt, Canadian Partnership Against Cancer: Synoptic Reporting Tools Project Evaluation).

Finally, implementation teams were required to mitigate barriers to SRT implementation and use, which ranged from general resistance to change to technological barriers (*e.g.*, computer availability). In both the mammography and endoscopy cases, many key informants felt that many of the perceived barriers could have been addressed by improved change management practices. In the cancer surgery case, key informants discussed the concerted efforts the implementation team made to remove obstacles to use and how these efforts facilitated SRT use: ‘[I was reluctant] the first couple of days because I said ‘I am not a computer person,’ but they made it easy’ (Physician #3).

### Champions and respected colleagues

Across all cases, the existence of respected, trusted clinical colleagues championing the initiative was critical to implementation and to clinicians’ acceptance and use of the tools. These champions played key roles in achieving buy-in for implementation (at all levels) and facilitating a credible implementation process. In the mammography case, key informants perceived the existence of local champions to be the biggest factor for radiologist buy-in and use, with the lack of a local champion perceived to impede implementation and use: ‘[We] need champions at the districts, especially [radiologists] … the biggest factor for radiologists, if local leaders wanted the system and supported it, it went well’ (Team member #2). In both the endoscopy and cancer surgery cases, the well-respected clinical colleagues who were leading and championing the initiatives were instrumental to facilitating use of the tools, despite many challenges with SRT use (either due to the tool itself or the way in which it was implemented). In fact, nearly all surgeons in the cancer surgery case indicated it was their respect for and trust in a clinical colleague that influenced their decision to use the SRT: ‘I trust [Dr. Z]’ (Physician #6).

These individuals’ influence extended beyond their clinical colleagues, facilitating the acquisition and leveraging of organizational resources and development of policy. In both the mammography and endoscopy cases, the influence of highly-respected clinicians who were leading and championing the initiatives was integral to developing and enacting policy with respect to using the SRTs for screening purposes.

### Administrative and managerial support

Key informant and document data indicated that all cases had strong support from senior administrators and executives at the organizational and health system levels (*e.g.*, health regions, government). These individuals perceived value from a quality improvement perspective, with SRTs aligning with higher-level strategic priorities and directions. Support and buy-in from senior administrators was fundamental to the teams’ abilities to implement these tools across different hospitals and their various organizational policies and infrastructure. For instance, in the endoscopy case, high-level support helped ensure the team acquired the necessary resources to achieve implementation: ‘[We] would put forward the budget request and the [Department of Health and Wellness] would honour that budget request and we were resourced in what we needed to do’ (Team member #2). In the mammography and endoscopy cases, support from system-level administrators ultimately led to the enactment of policy requiring clinicians to use the SRTs for all screening investigations.

The level of support from middle managers and department/unit heads varied across the cases though had a considerable impact on the implementation experience—both for the implementation teams as well as clinician users. In the mammography and endoscopy cases, support from middle managers was low in some hospitals. Lack of managerial support was largely related to their low involvement and input during implementation, the introduction of new roles/tasks with no additional resources to carry out this new work, lack of IT integration (which added to the workload in many departments), and their limited understanding of the tool’s value. This low support was expressed by one participant in the endoscopy case, who said, ‘Everyone says these systems will be cost-savings and time-savings, but I don’t believe [this tool] is either’ (Organizational member #2). Conversely, managers were highly supportive of the cancer surgery case, helping the team leverage resources (*e.g.*, time, expertise) and navigate the organizational and socio-political landscape. Key informants stated they felt the team accomplished such a high level of support via widespread stakeholder engagement and personal contact with stakeholders throughout the system.

### Innovation attributes

Characteristics of the SRTs undoubtedly influenced their implementation and use. Specifically, the complexity/simplicity of the input system and resulting end report, the relative advantage the tool had over existing practices, and the extent to which the tool aligned with individual, departmental, and organizational values, interests, and prior experiences (*i.e.*, compatibility) all contributed to individuals’ perceptions of the SRTs and their willingness to implement and use them.

The ease of use of the tools (including the end report) varied across cases, with clinician users in the endoscopy and cancer surgery cases more apt to describe the SRT as user-friendly than users in the mammography case. Nearly all endoscopists in the endoscopy case, for instance, stated that the SRT was easy to learn and use, and that it saved time when compared to dictating: ‘As far as using the computer program, it’s basically very intuitive. There are one or steps that aren’t but they are simple once you get the trick of it’ (Physician #4). Similarly, most surgeons in the cancer surgery case indicated the tool was relatively easy to use, though took more time to complete than traditional narrative (dictated) reporting. Despite this, most users expressed some dissatisfaction with certain features of the tools (*e.g.*, quantity of data elements) and/or the end report (*e.g.*, length) though these views were not universal. Technical and accessibility issues (*e.g.*, login difficulty) were also a large source of frustration for users, especially when they were unable to access technical support in a timely manner to resolve them.

Many radiologists in the mammography case did not find the SRTs user-friendly and those who refrained from using the tool expressed that it was not advantageous compared to current reporting practices. One particular issue that nearly every key informant, outside of the implementation team, discussed was the end report that was generated. This report was perceived as inadequate and confusing to review, with key informants from multiple levels of the system stating they did not view it as a true synoptic report. These issues are exemplified in the following remarks:

‘Whatever system is out there, it can’t make our job more difficult, the report that comes out has to be the same or better, not worse, uh and the people that are reading those reports have to understand what we are saying and in this case none of those are true’ (Physician #3).

Across cases, clinicians emphasized that, in an already-overburdened environment, using a SRT (or any new tool in practice) must be as easy as what they currently do, at least after the initial learning curve.

Data across multiple sources strongly indicated that synoptic reporting aligned with organizational and system values, directions, and priorities. Perceived value related to clinical utility, organizational efficiencies, and the potential for performance monitoring and quality improvement. Endoscopists and surgeons perceived that the SRTs were compatible with their individual and professional values, despite any specific issues with the tools themselves. Most identified clinical benefits to using SRTs, including standardization and timeliness of information, improved communication with other care providers, and enabling best practices. These views are illustrated by one clinician user in the cancer surgery case, who stated:

‘I think that we probably all agreed that in the traditional system of just dictating operative notes, there is great variability of information that is provided, uhm, it created problems for communication for what was found, oncologists trying to figure what we did or didn’t do and, uhm, also from a quality assurance perspective. You know, there are certain things that should be in there and [the synoptic report] can help achieve addressing those issues. It is just a good thing’ (Physician #1).

### Use of SRTs across cases

The extent of SRT use was revealed via key informant interviews and document analysis (mammography case, endoscopy case) and a brief review of one SRT database (cancer surgery case). Table 7 describes use across the cases.

**Table 7:** **Use of the synoptic reporting tool (SRT) by case, at the end of data collection (February 2012)**

**Case**	**Data source(s)**	**Extent of use**
Mammography case	Key informant interviews, documents	● All radiologists in the province use the screening SRT; use of this tool has been ‘strongly recommended’ by government since 2008 in response to a provincial policy related to national mammography accreditation
		● Radiologists in three districts have chosen not to use the diagnostic SRT for their reporting of diagnostic mammography
Endoscopy case	Key informant interviews, documents	● All endoscopists in the province use the SRT for screening colonoscopies; use of the tool is required for participation in the screening program
		● Most endoscopists in one district use the SRT for all endoscopic procedures; a district-wide policy was in the process of being implemented
		● Most endoscopists in the eight remaining districts do not use the SRT for diagnostic colonoscopy
Cancer surgery case	Key informant interviews, database review	● 4 of 4 breast surgeons in the two tertiary care centres consistently use the SRT to report breast cancer surgeries^a^
		● 3 of 4 colorectal cancer surgeons at the tertiary care centre consistently use the SRT to report colorectal cancer surgeries
		● 1 of 2 general surgeons in the community hospital consistently uses the SRT to report breast and colorectal cancer surgeries

^a^The review of the database revealed more synoptic reports than actual breast cancer surgeries, indicating some surgeons use the SRT to also report benign breast surgeries.

## Discussion

This study examined the key multi-level factors that influenced the implementation and use of SRTs in three cases of cancer care in one Canadian province. Cross-case analysis revealed five common factors that were particularly influential across the cases studied. That these factors transcended the different contexts (settings, timing, and individuals) demonstrates their importance to gaining buy-in and support for implementation, promoting a sense of ownership for implementation, acquiring and leveraging resources (human and fiscal) to make the implementation a reality, and providing people with reasons to change and the tools to help them succeed.

The theoretical perspectives that guided this study emphasize various multi-level influences on innovation implementation. The five factors that were common across cases were represented across the three perspectives, either explicitly as a construct or by encompassing some of the same concepts as the constructs embody (see Table 8). As presented in Table 8, many of the constructs of the organizational framework of innovation implementation [45] were salient to SRT implementation and use across all cases. Similarly, the propositions put forward by Kitson [46] (see Table 1) were all germane to the cases studied.

**Table 8:** **Key factors influencing synoptic reporting tool (SRT) implementation and use and their relationship to the theoretical perspectives (1 = Promoting Action on Research Implementation in Health Services; 2 = Organizational framework of innovation implementation; 3 = Systems thinking / change)**

**Influencing factors**	**Relevant theoretical perspective(s); construct(s)**	**Relationship to theoretical perspective(s)**
*Common factors*		
Stakeholder involvement	3; local autonomy, (re)negotiation, resources	Key stakeholder involvement influenced SRT implementation and use, with high involvement critical to navigating the healthcare system, building a sense of local ownership, and acquiring moral and material support for implementation.
Managing the change process	2; implementation policies and practices, implementation climate	Employing policies and practices to manage resistance and other barriers to SRT implementation and use, communicate about the SRT and its implementation, and provide training and support were important parts of managing the change process.
Administrative and managerial support^a^	2; management support	In organizations wherein administrative and managerial support were high, implementation went smoother and the experience tended to be better for end users; where support was low, the reverse occurred.
Champions and respected colleagues	2; innovation champions	Respected colleagues who championed the SRT were instrumental to clinicians’ decisions to use the SRTs and to continue using, even in settings wherein ongoing challenges and frustrations were prevalent.
Innovation attributes	2; innovation-values fit	Innovation-values fit is akin to one of the concepts—compatibility—encompassed in the key factor innovation attributes. High compatibility or ‘fit’ existed between SRTs and individual, organizational, and system values, interests, and priorities.
	3; nature of knowledge	Implementation and use was influenced by the way in which participants understood the SRTs. Individuals’ understandings of the nature and characteristics of the SRTs were depicted as attributes of the innovation, specifically complexity, relative advantage, and compatibility. When individuals believed that the SRT held value and would (at least eventually) be better than the practice it replaced, they were much more apt to support its implementation and use.
*Distinct factors*		
Implementation approach	*Neither*	In the endoscopy and cancer surgery cases, SRT implementation and use were influenced by the tool’s positioning in the healthcare system (*i.e.*, part of a screening program; pilot project) and the related implementation approach (*i.e.*, top-down, policy driven; ground-up). Neither of the theoretical perspectives specifically addresses how these factors might affect innovation implementation.
Project management	*Neither*	In the endoscopy case, SRT implementation was impeded by suboptimal project management, specifically related to the tool’s implementation. Neither of the theoretical perspectives specifically addresses project management as an important influence on moving knowledge into practice, though task-based ‘facilitation’ [64] may include some of the project management practices encompassed in this factor.
Resources	2; financial resource availability	Limited financial resources, including financially dependent resources (*e.g.*, acquiring personnel), was deemed a key constraining factor in the mammography and cancer surgery cases. Limited resources affected change management practices (mammography) as well as information technology work to update/refine the SRT (mammography) and adapt the SRT to the Nova Scotia environment (cancer surgery).
Culture	1; context (culture)	In the mammography case, SRT implementation and use were facilitated by the program’s strong quality improvement culture.
Leadership	1; context (leadership)	In the mammography case, SRT implementation and use were facilitated by consistent and effective leadership; the leaders, who have largely remained stable over two decades, were effective at building a dedicated team and acquiring the resources for SRT implementation.
Monitoring and feedback mechanisms	1; context (evaluation)	SRT implementation and use in the mammography case were facilitated by ongoing monitoring and feedback mechanisms at multiple levels of the healthcare system (*e.g.*, clinicians, health districts, government).
Components of the healthcare system	3; *no specific construct*	In the endoscopy and cancer surgery cases, SRT implementation was impeded by structural, infrastructural, and/or socio-historical components of the healthcare system. ‘Systems’ thinking / change views the healthcare system as an interdependent, social system wherein the movement of knowledge into practice is impacted by the larger system’s characteristics (*e.g.*, relationships across the system, historical interactions, and so on).

^a^Administrators = executive officers, directors, and senior management at the Department of Health, health district, and hospital levels; management = managers and heads of organizational departments and units.

One of the constructs of the PARiHS framework—context—influenced SRT implementation in the mammography case, but less so in the other cases. Interestingly, neither the construct of evidence (even defined broadly through PARiHS) nor facilitation (as conceptualized by PARiHS) was an influential factor in SRT implementation and use. Most key informants did not discuss the evidence for synoptic reporting as being a factor in their decisions to adopt and/or use the tools. When evidence was discussed, the source of evidence was most often data from other jurisdictions (*e.g.*, local evaluations, verbal experiences with use) than the scientific literature. Related to facilitation, the implementation teams in each case were responsible for supporting affected individuals before and during SRT implementation. However, key informant and documentary data did not indicate a need for facilitation as described by PARiHS, specifically for a dedicated, trained individual to work with the team ‘to construct a programme of change that meets the individual and team’s learning needs’ [44] (pg. 10). Rather, the data emphasized particular facets of implementation processes that dedicated teams must attend to and engage in. Thus, aspects of facilitation were clearly encompassed in the findings related to stakeholder involvement and managing the change process. Broadening our understanding of facilitation as a team or organizational construct wherein many individuals can adopt strategies and activities that facilitate implementation—versus a position filled by a trained individual—may provide a more nuanced understanding of facilitation in innovation implementation.

The key factors influencing implementation and use in the endoscopy and cancer surgery cases were quite similar. This is perhaps not surprising given the initiatives took place at approximately the same time in the same province and involved some of the same stakeholders at the system and user levels. However, the specific influence of those factors (*i.e.*, facilitating or impeding) often differed between cases, with the implementation approached quite differently with respect to many of the factors. The cases also had many differences (*e.g.*, resource characteristics and availability) with entirely different implementation teams. That commonalities existed in spite of the differences, and that many of the same overarching factors were also common to the mammography case, strengthens our findings and helps to extend and refine theory in the area of innovation implementation in health care. Specifically, the findings add novel insights into several important issues that are under-developed in the existing literature in this area. First, they revealed the important role that individuals at the middle-level of organizations (*e.g.*, middle managers, department/unit heads) play in implementation efforts. Indeed, the data demonstrated that these individuals can facilitate innovation implementation by demonstrating their moral support for implementation (*e.g.*, involving staff in pre-implementation planning); exerting their authority over existing departmental policies, priorities, and resources (*e.g.*, providing on-the-ground resources, such as staff time, to facilitate implementation); and influencing the development of policy related to the innovation and its implementation (*e.g.*, championing the innovation with senior administrators who can develop and enact organizational policy). Their influence, however, can be positive or negative—that is, these individuals can allocate resources to support implementation or ensure that implementation is something that is carried out ‘off the side of one’s desk.’

Second, the findings revealed that the interpersonal aspects of change have a considerable influence on innovation implementation and use. For instance, the findings clearly showed the facilitating influence of involving stakeholders early in implementation planning and from multiple levels of the healthcare system, not simply clinician users. Quite simply, stakeholders who felt they were highly involved in the implementation were more willing to help the team navigate the implementation at their respective organizations, and to provide organizational and departmental resources (*e.g.*, staff time and expertise). Those with low involvement were, by and large, resistant to SRT implementation—despite speaking highly of SRTs in general—and cited numerous reasons for opposing the SRT or its implementation. This finding has important practical implications because individuals can make choices and frame issues in ways that influence others and thus have consequences for implementation. Moreover, the data suggest that stakeholders should include not only clinician users and senior administrators, but also other organizational members who can affect, and are affected by, the implementation. Indeed, many of the ‘micro-processes’ of implementation have to be negotiated with local stakeholders [65]; these individuals have the knowledge and expertise, and oftentimes access to local resources, to provide solutions that are workable and sensitive to local conditions and capacities.

This study has a number of strengths. First, the multiple strategies employed to increase rigor enhance confidence in our findings. Second, there was a high level of participation across units of analysis for all cases. Six individuals did fail to respond in the mammography case; though the reason(s) for this is speculative, it may have involved clinician time/interest or a perception that he/she had nothing of value to share. Indeed, these six individuals were radiologists at institutions wherein the SRTs had been in use for more than a decade. It is plausible that the SRTs were in place when they commenced their practice and therefore they felt they had little insight into their implementation and opted not to respond to the invitation.

This study does have limitations. First, this study was undertaken in one jurisdiction only. Given that the structure and socio-political context of healthcare systems vary, this may limit the applicability of findings to other jurisdictions. Nonetheless, healthcare systems generally have a number of defining features, including a wide range and diversity of stakeholders, complex governance and resourcing arrangements, and high degrees of professional autonomy of many of its staff [66], which should increase the applicability of these findings in other health systems. Moreover, the sampling strategy ensured that the cases varied on key constructs believed to influence innovation implementation, and the healthcare delivery system in the province differed considerably across the implementation timeframes. These differences across cases also facilitate the applicability of findings to other contexts. A second limitation pertains to the mammography case, wherein a number of key informants stated it was difficult to remember what happened during the implementation period. Therefore, the data are subject to issues of recall. Of the key informants who were involved during the initial implementation efforts, however, their recollections of people and events during that time did not differ considerably from one another.

## Conclusions

Key factors at multiple levels of the health system affected SRT implementation and use. The five factors common across cases were: stakeholder involvement, managing the change process, administrative and managerial support, champions and respected colleagues, and innovation attributes. The key role of interpersonal level factors across cases, despite differing characteristics and contexts, and their relationships to gaining and maintaining both moral and material support for innovation implementation have significant implications for individuals and teams who are responsible for implementing changes in healthcare settings. Indeed, the findings revealed that positive relationships can counterbalance many negative contextual factors—thus, the early engagement of key stakeholders across multiple levels of healthcare organizations and systems may be fundamental to implementation efforts and to supporting the consistent and committed use of an innovation. The findings also demonstrate the importance of a multi-level contextual analysis to gaining both breadth and depth to our understanding of innovation implementation and use in health care. Recent conceptual work on implementation in health care [67] and evidence-based practice [68] supports the need for this type of analysis.

## Additional file

Additional file 1:
**Organization of health care in Nova Scotia.**

## Abbreviations

PARiHS: Promoting Action on Research Implementation in Health Services framework
SRT: Synoptic reporting tool
