# Supplementary material for: Multi-level factors influence the implementation and use of complex innovations in cancer care: a multiple case study of synoptic reporting
Source: Implement Sci. 2014 Sep 16;9:121. doi: 10.1186/s13012-014-0121-0 (PMC4173056; doi:10.1186/s13012-014-0121-0)
Supplement: Additional file 1: — Organization of health care in Nova Scotia. [file 13012_2014_121_MOESM1_ESM.doc]

**Additional file 1**

**Organization of health care in Nova Scotia**

Health care delivery

The provincial health care delivery system is comprised of nine district health authorities (DHA) and the IWK Health Centre (IWK), the province’s consolidated women’s and children’s hospital in Halifax. The health authority structure is legislated under the Health Authorities Act and all DHAs and the IWK are legal entities. Altogether, there are 34 acute care facilities in the province operated by the nine districts and the IWK. Twenty-eight of these facilities are outside of the largest metropolitan region.

Under the current legislation, the DHAs receive funding from the Department of Health and Wellness (DoHW; formerly Department of Health) but have the authority and autonomy to plan, manage, deliver, monitor, and evaluate health services within their jurisdiction. However, unlike most Canadian provinces, the DoHW does not have performance or accountability agreements in place with DHAs (or individual hospitals) to facilitate their participation in provincial transformation initiatives and reporting/monitoring.

*Historical context*. Prior to 1996, 36 local hospital boards were responsible for health care delivery in the province and governed the day-to-day administration of local hospitals. In 1994, under a Liberal government, the province began a reconfiguring of its health system (this was in response to a 1989 provincial Royal Commission on Health Care). The justification for this reconfiguring was cost containment and greater accountability/citizen participation. By 1996, the 36 hospital boards were amalgamated into four regional health boards (Central, Western, Northern, Eastern).

In 1999, the Conservative government campaigned, in a general provincial election, that it would eliminate the four regional health boards and replace them with nine DHAs. The justification for this proposed expansion was cost containment and greater accountability/citizen participation. In January 2001, under a majority Conservative government, the four regional health boards were expanded into nine DHAs (the current structure) despite recommendations from an expert panel that, while the restructuring had experienced some challenges with implementation, the regional health board structure should not be abandoned and that the system could not afford another major structural change. These DHAs assumed the same responsibilities as the prior regional health boards but with smaller catchment areas.

Provincial program organization

The DoHW funds nine provincial programs in Nova Scotia. The organization of these programs and their governance model are directly relevant to the implementation of synoptic reporting in two of the cases studied: the Colon Cancer Prevention Program (which is hosted by Cancer Care Nova Scotia, one of the provincial programs) and the Nova Scotia Breast Screening Program (which is one of the provincial programs). Since 2004, a provincial program model has been in place that outlines the mandate of these programs:

- Act in an advisory capacity to the DoHW
- Recommend service delivery models (i.e., advise care providers)
- Develop draft standards
- Educate and communicate about standards and best practices
- Monitor approve standards
- Work with provider organizations to ensure implementation
- Participate in program evaluation

This mandate is significant in that it underscores the advisory role of the provincial programs. The program model separates policy development and standard setting from care delivery: provincial programs are not directly responsible for providing care (this is the role of DHAs and the IWK) and they generally lack authority for monitoring and non-compliance.

While each program is responsible for managing its day-to-day operations, the programs are not separate legal entities. This means they cannot be direct employers or hold funds in their own right. The DoHW has contracts and/or memoranda of understanding with host organizations, usually academic healthcare centres, wherein these organizations support provincial program operations (e.g., office space/overhead, employee services, and information technology [IT] services). All contracts involving provincial programs are entered into under the authority of the Crown and all assets of provincial programs, including data, are owned by the DoHW.

Information technology organization and infrastructure
There is no single IT platform implemented across Nova Scotia. Eight DHAs have a range of clinical, financial, and administrative software applications hosted on a common Meditech Client-Server platform. This single vendor solution allows these largely rural DHAs to accommodate more primary and secondary healthcare requirements. The IWK operates on Meditech Magic platform. Capital Health (DHA 9) has adopted a best-of-breed (multi-vendor) approach to its clinical applications, with no Meditech products. This approach was likely due to the fact that Capital Health provides a more complex mix of tertiary and referral care, includes the province’s leading academic medical centre, and engages in much more medical research/clinical trials. Many of Capital Health’s clinical solutions were acquired from some of the leading vendors at the time of procurement.

The DoHW established the Health Information Technology Services Program of Nova Scotia (HITS-NS) in 2006 as the centralized provincial service delivery organization for the province’s shared IT services. HITS-NS is funded by the DoHW but is a separate entity hosted at the IWK. This decision was based on the fact that HITS-NS holds a great deal of patient and institutional information, and the DoHW felt this information was best held by a quasi-independent organization.

HITS-NS oversees the expanding inventory of clinical, financial, and administrative software applications for DHAs1-8 (all hosted by HITS-NS on a common Meditech Client-Server platform) as well as some applications for both IWK and Capital Health. HITS-NS also has operational oversight of a number of additional IT services, including the province’s Picture Archiving and Communications Systems, Primary Healthcare Information Management systems, and TeleHealth video-conferencing.

In essence, HITS-NS provides operations support for provincial IT systems. The organization is not tasked with the role of identifying, developing, and implementing new IT systems. Rather, the organization is charged with the responsibility of ensuring that existing and proposed IT systems follow defined standards and interface to other systems using acceptable protocols and procedures. HITS-NS’ role in ‘accepting’ any new IT system and helping with the technical work is based on a DHA’s approval of implementing the system.

There is no provincial plan on how to best leverage information management/IT systems in Nova Scotia.[[1]](#footnote-2) As explicated by a key informant at the DoHW, the rapidly expansion of technology and the subsequent demands on HITS-NS services has meant that the organization has not grown in an organized or planned way. The lack of a clear provincial plan is a significant barrier in the development of the province’s IT infrastructure.

Privacy and data sharing environment

Around the early to mid-1990s, governments at provincial and territorial levels in Canada began to enact laws to regulate the collection, use, and disclosure of personal information in the public sector. These laws followed increasing public concern about the privacy of personal information. Thus, in alignment with other jurisdictions, Nova Scotia enacted the *Freedom of Information and Protection of Privacy Act* (*FOIPOP*) in 1993 to regulate access to and privacy of personal information held by public bodies, including government departments, provincial agencies, boards, and commissions, universities, DHAs, and hospitals. This “personal information” included an identifiable individual’s healthcare history.[[2]](#footnote-3)

However, during implementation of synoptic reporting in the three cases studied, there was no legislation in Nova Scotia to specifically protect the privacy of personal health information. Instead, personal health information was managed according to more than 40 different pieces of legislation, including the *FOIPOP Act*, *Hospitals Act*, *Health Protection Act*, and the Federal *Personal Information Protection and Electronic Documents Act (PIPEDA)*, which falls under the mandate of Industry Canada. The rules for providers, health records, and facilities were not always consistent across Acts.[[3]](#footnote-4) For instance, some of the Acts do not use a consent-based model to collect and use personal information, while others do. *FOIPOP*, for example, authorizes public bodies to collect personal information where authorized by law or where necessary for the operation of the body’s programs. These bodies may use or disclose information without specific consent for the purpose(s) for which it was obtained or a similar purpose. *PIPEDA*, however, maintains that, in general, explicit consent should be sought for the collection, use, and disclosure of sensitive information, including secondary uses of identifiable health information.

Moreover, this legislative structure was largely developed in an era of paper-based records and was considerably challenged by electronic personal health information. For example, applying legislation on informed consent to the collection, use, and disclosure of personal health information can be challenging in the context of electronic medical records and other clinical systems. Specifically, it may be next to impossible to obtain truly informed consent for uses and disclosures of personal health information given that all future uses of information in electronic medical records and other clinical care databases cannot be foreseen when the information is initially entered into the system.[[4]](#footnote-5)

Alongside this legislative context, in January 2005, the Nova Scotia Deputy Minister of Health endorsed a *Pan-Canadian Health Information Privacy and Confidentiality Framework*.[[5]](#footnote-6) The *Framework* was meant to be a tool to inform and influence privacy legislation processes within provincial and territorial jurisdictions, and it set out a number of core provisions that attempted to strike a balance between protecting the privacy and confidentiality of individual health information and enabling the flow of information to support effective health care delivery, management of the health system, and an interoperable electronic health record. For instance, the *Framework* advises there are circumstances wherein explicit consent should *not* be required for the use or disclosure of personal health information, as long as certain conditions are met (e.g., legislation authorizing use and disclosure, or a privacy impact assessment subject to review by a Commissioner or Review Officer). Such circumstances include: ensuring quality of standards of care within the trustee/custodian organization (e.g., disclosure for quality of care committees or similar bodies) and planning and management of the health system.

In addition, each health care facility has its own policies and procedures related to privacy, security of personal health information, and data integrity. While they may be similar, the introduction of any new clinical database and the transfer of personal information across databases required that the database be implemented in such a way that it adhered to the policies of the particular institution. Similarly, given that the synoptic report is the legal equivalent to the dictated report, the final report must meet the legal requirements of health records, privacy, and health information management departments/offices at each institution.

In December 2010, Nova Scotia passed legislation, the *Personal Health Information Act*, to protect the privacy of personal health information. This Act came into effect May 2013.

1. Corpus Sanchez International (CSI) Consultancy Inc. *Provincial Health Services Operational Review Final Report: System Level Findings & Overall Directions*, December 2007. [↑](#footnote-ref-2)
2. Freedom of Information and Protection of Privacy Act. *1993, c. 5, s. 1.* [↑](#footnote-ref-3)
3. Nova Scotia Department of Health. *Personal Health Information Legislation for Nova Scotia: Discussion Paper*, 2008. [↑](#footnote-ref-4)
4. For further discussion of the consent challenge in the EHR context, see: Work, F. *Issues with respect to the Electronic Patient Record,* Office of the Privacy Commissioner of Alberta, October 2002. [↑](#footnote-ref-5)
5. Health Canada. *Pan-Canadian Health Information Privacy and Confidentiality Framework*. January 27, 2005. Available online at: http://www.hc-sc.gc.ca/hcs-sss/pubs/ehealth-esante/2005-pancanad-priv/index-eng.php. [↑](#footnote-ref-6)
